# Assessment of the financial toxicity in patients with differentiated thyroid cancer: a cross-sectional study in western China

**DOI:** 10.3389/fpubh.2025.1391744

**Published:** 2025-05-15

**Authors:** Zhou Jun, Chen Qiaoling, Li Qianqian, Jiang Hua, Lei Yu, Yang Xue, Liu Lemei, Li Fanmin

**Affiliations:** ^1^Department of General Practice, The People’s Hospital of Leshan, Leshan, China; ^2^Department of Operating Room, The People’s Hospital of Jiajiang, Leshan, China; ^3^Sichuan Cancer Hospital and Institute, Sichuan Cancer Center, School of Medicine, University of Electronic Science and Technology of China, Chengdu, China; ^4^Department of Nursing, The People’s Hospital of Leshan, Leshan, China

**Keywords:** financial toxicity, financial burden, differentiated thyroid cancer, psychological distress, cancer

## Abstract

**Objective:**

Using the Comprehensive Score for Financial Toxicity (COST) tool to measure financial toxicity (FT) among differentiated thyroid cancer (DTC) patients in China and investigate the association between FT and psychological distress.

**Methods:**

We carried out a cross-sectional investigation of individuals who had survived DTC in two tertiary medical facilities. The assessment of FT was performed using the Chinese version of the COST tool. The National Comprehensive Cancer Network (NCCN) Distress Thermometer (DT) was used to measure psychological distress. A multivariate logistic regression model was constructed to identify factors related to FT, and the Pearson correlation was used to evaluate the association between COST and DT scores.

**Results:**

Out of the 207 patients who participated in this study, the average COST score was 16.3. Notably, the prevalence of financial toxicity was 47.8% (95% CI: 41% ~ 54.7%) of the patients. Of these, 22.7% (47/207) were mild FT, 23.7% (49/207) were moderate FT, and 1.4% (3/207) were severe FT. Four variables were found to be associated with increased FT in the logistic regression model, younger age (odd ratio [OR], 4.52; *p* = 0.003), lower educational level [OR], 1.13; *p* = 0.040, uninsured (odd ratio [OR], 6.53; *p* = 0.028), had lower household income (odd ratio [OR], 6.34; *p* = 0.037), and advanced cancer (odd ratio [OR], 2.99; *p* = 0.034). Furthermore, the Pearson correlation revealed a mild correlation between financial toxicity and psychological distress (r = −0.53, *p* < 0.001).

**Conclusion:**

In this study, the prevalence of FT in DTC patients was 47.8%. FT was associated with younger age, lower educational level, uninsured, had lower household income, and advanced cancer. Clinicians should identify patients by predictors early and conduct psychological interventions.

## Introduction

The incidence of thyroid cancer continues to rise worldwide. Thyroid cancer (TC) is the most prevalent endocrine malignancy, accounting for 3–4% of all cancers, with an incidence rate ranking ninth among all cancers in 2020 (1, 2). China has a high incidence of thyroid cancer, new cancer cases account for 1/4 of the world’s total, and the incidence rate has increased significantly and tends to be younger patients (1, 3).

Thyroid cancer can be classified into the following four types according to the pathological type of the disease: papillary thyroid cancer (PTC), follicular thyroid cancer (FTC), and medullary thyroid cancer (MTC) and undifferentiated carcinoma (ATC). Among them, PTC and FTC were classified as DTC (DTC), accounting for more than 90% of all thyroid cancers (2). There are various treatment options for different pathological types of thyroid cancer, and surgery is the preferred treatment option (4). Although the five-year survival of thyroid cancer patients is as high as 98.2% (2), they may bear a substantial financial burden due to the diagnosis and treatment of cancer (5). Among cancer survivors, thyroid cancer has been reported to be at higher risk of financial hardship and bankruptcy compared with colon, breast, lung, and prostate cancer (6, 7). Currently, most studies on financial burden involving thyroid cancer patients primarily come from developed Western countries. In comparison, China has a lower per capita income, leading to a heavier economic burden for young individuals seeking medical treatment. Although the mortality rate of thyroid cancer is not high, the affected population consists mostly of young individuals who may have limited savings. Undergoing treatment for thyroid cancer can result in considerable FT for this demographic, which can impact their post-operative psychological well-being and overall quality of life (7). Therefore, given the context of China, it is essential to understand the level of FT experienced by this specific group of individuals.

In recent years, the cancer-related financial burden has gradually attracted attention in the field of oncology. Financial toxicity (FT) was defined as the objective financial burden and subjective financial distress of cancer patients due to treatments using innovative drugs and concomitant health services, similar to side effects such as nausea and vomiting (8). FT can be influenced by demographics, economic status, disease, treatment, etc. Taking into account differences in cultural background and health systems, the influencing factors of FT may vary among countries (9). Previous research has demonstrated that FT endangers patients’ mental health and decreases their quality of life and treatment compliance (9, 10). Therefore, it is necessary to correctly measure the FT of survivors in the early stage. Prior studies have suggested that FT should be assessed using patient-reported outcomes (PROs), as they are more effective in capturing cancer survivors’ personal thoughts, concerns, and perspectives compared to numerical data or external observations (11). The Comprehensive Score of Financial Toxicity (COST), developed and validated by De Souza et al. (12), has been validated as an effective measure in medical oncology patients across multiple countries and cancer types. Although individual studies may have included DTC samples, there is no specific research conducted in China targeting this population. Therefore, it is necessary to investigate the severity of FT and potential risk factors among patients with DTC by utilizing the COST. In this study, we aimed to apply the COST instrument to the thyroid cancer setting and to identify factors associated with FT in this population.

## Materials and methods

### Study design

We conducted a cross-sectional study in two tertiary hospital in China between June 2022 and April 2023. Patients were eligible to participate if they (i) were >18 years, (ii) with pathologically diagnosed DTC, (iii) had undergone surgery, and (iv) consented to participate in the survey. The excluding criteria contained: (i) currently being treated for another malignancy, (ii) participating in other clinical trials, and (iii) unable to read, understand and speak Chinese.

We conducted face-to-face interviews with all eligible inpatients and provided them with questionnaires to complete. To gain a comprehensive understanding of the sociodemographic and socioeconomic characteristics of the patients, we designed a general information questionnaire. We extracted information on the clinic data of the patients from the electronic medical records from the Hospital Information System (HIS).

FT was measured using the COST tool (Chinese version), which has been shown to have good reliability and validity (13). The total score ranges from 0 to 44, with lower scores indicating more severe FT in patients. According to the FT grading system, a COST score > 26 indicates no FT (grade 0), 14–25 indicates mild FT (grade 1), 1–13 indicates moderate FT (grade 2), and COST score = 0 indicates severe FT (grade3) (14). This grading system is based on the original development study of the COST scale by De Souza et al., serving as a standard for assessing financial toxicity in cancer patients (14). The Cronbach’s α of the Chinese version of COST is 0.891.

We also measured psychological distress using the National Comprehensive Cancer Network (NCCN) Distress Thermometer (DT). The total score ranges from 0 (no distress) to 10 (great distress); A score of 4 has been determined to be the cut-off score for moderate psychological distress and the trigger for psychological assistance referral (15).

### Sample size determination

The sample size was calculated using the single population proportion formula, considering the following assumptions and taking a prevalence of 78% which was reported in a systematic review of FT in cancer survivors in China (16).


$$n=\frac{{\left(Z_{1-\alpha }/2\right)}^{2}p(1-p)}{d^{2}}$$

In the above formula, *n* = the desired sample size, *p* = the prevalence of FT = 78%, Z1-*α*/2 = critical value at 95% confidence level (1.96), *d* = the margin of error = 6%, *n* = (1.96)^2^*0.78*(1–0.78) / (0.06)^2^ = 183. For possible non-response during the study, the sample size was increased by 10%, so the final total sample size was: *n* = 183/(1–10%) = 203.

### Statistical analysis

Descriptive statistics were used to describe patients’ characteristics. Chi-square test, Fisher’s exact test, student’s *t*-test, or Wilcoxon rank sum was used in univariate analysis as appropriate. We used multiple logistic regression analysis to determine factors associated with the composite measure of financial toxicity. Multivariable regression analysis included significant covariates identified in univariate analysis (*p* < 0.05) and covariates thought to be of clinical significance.

Pearson correlation method was used to assess the correlation between COST and DT scores. If the coefficient (r) is 0.20 to 0.39, it is considered a mild correlation; 0.4–0.59 is a moderate correlation, 0.60–0.79 is a strong correlation, and ≥0.80 is a very strong correlation (17). All statistical analyses were performed with SPSS 26.0 (IBM, NY, USA). Statistical significance was considered with a *p* < 0.05.

## Results

### Patient participation and characteristics

**Figure 1:**
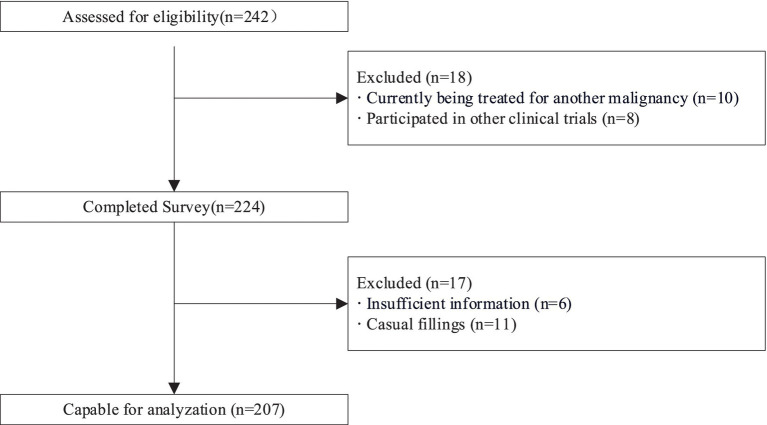
Patient recruitment process.

Recruitment process is presented in Figure 1. The study cohort was accessed between June 2022 and April 2023. A total of 242 questionnaires were distributed, 18 patients did not meet our inclusion criteria as they had participated in other clinical trials or treated for another malignancy, out of which 224 patients agreed and completed the questionnaire. After excluding 17 invalid questionnaires, 207 people were capable for analysis, with a response rate of 85.5%. In this analysis, the median age was 52.4 years (range: 20–67 years), 57.0% of patients had social insurance, 20.7% had commercial insurance, and 14.0% had both. Most of them (77.1%) had tumor stage I ~ II. Among these patients, 27.0% had an annual household income below 60,000CNY, 26.6% were between 60,000CNY and 120,000CNY, 18.8% were between 120,000CNY and 200,000CNY, 13.6% were between 200,000CNY and 300,000CNY, and 14.0% were above 300,000CNY (1CNY = 0.14USD, as of 2023-12-06). Table 1 shows the demographic and medical characteristics of the patients.

**Table 1:** Patients characteristics by COST score (*N* = 207).

Characteristics	*N*	COST≥26 (*n* = 108)	COST<26 (*n* = 99)	*p*
Sex				0.184
Male	70	32 (45.7%)	38 (54.3%)	
Female	137	76 (55.5%)	61 (44.5%)	
Age				<0.001
20 ~ 40	92	42 (45.6%)	50 (54.4%)	
40 ~ 55	69	30 (43.5%)	39 (56.5%)	
>55	46	36 (78.2%)	10 (21.8%)	
Place of residence				0.025
Urban	115	68 (59.1%)	47 (40.8%)	
Rural	92	40 (43.5%)	52 (56.5%)	
Marital status				0.047
Married	146	82 (56.1%)	64 (43.9%)	
Unmarried	35	19 (54.3%)	16 (45.7%)	
Divorced	16	5 (31.3%)	11 (68.7%)	
Widowed	10	2 (20.00%)	8 (80.00%)	
Education level				0.001
High school or blow (<9 year)	102	46 (45.1%)	56 (54.9%)	
Some college (9 ~ 12 year)	64	30 (46.9%)	34 (53.1%)	
College graduated or above (>12 year)	41	32 (78.0%)	9 (22.0%)	
Employment status				0.014
Employed	101	61 (60.4%)	40 (39.6%)	
Unemployed	79	31 (39.2%)	48 (60.8%)	
Retired	27	10 (37.0%)	14 (63.0%)	
Health insurance				0.001
Uninsured	17	6 (35.3%)	11 (64.7%)	
Social insurance	118	63 (53.4%)	55 (46.6%)	
Social + Commercial	72	39 (54.2%)	33 (45.8%)	
Household income per year (CNY)				0.001
<60,000	56	26 (46.4%)	30 (53.6%)	
60,000 ~ 120,000	55	28 (50.9%)	27 (49.1%)	
12,000 ~ 200,000	39	21 (56.4%)	18 (43.6%)	
20,000 ~ 300,000	28	20 (71.4%)	8 (28.6%)	
>300,000	29	25 (86.2%)	4 (13.8%)	
Out-of-pocket costs (CNY)				0.001
<6,000	80	56 (70.0%)	24 (30.0%)	
6,000 ~ 10,000	65	35 (53.8%)	30 (46.2%)	
>10,000	62	17 (27.4%)	45 (72.6%)	
Travelling time to hospital				0.172
<1 h	30	16 (53.3%)	14 (46.7%)	
1 ~ 3 h	90	46 (51.1%)	44 (48.9%)	
3 ~ 5 h	40	16 (40.0%)	24 (60.0%)	
>5 h	47	30 (63.8%)	17 (36.2%)	
Number of chronic disease				0.476
0	130	62 (47.7%)	68 (52.3%)	
1	48	28 (58.3%)	20 (41.7%)	
≥2	29	18 (62.0%)	11 (38.0%)	
Tumor stage				0.001
I ~ II	163	96 (58.9%)	67 (41.1%)	
III ~ IV	44	12 (27.2%)	32 (72.8%)	
Diagnosis time				0.248
<1 month	44	18 (40.9%)	26 (59.1%)	
1 ~ 6 month	108	59 (54.6%)	49 (47.4%)	
6 ~ 12 month	37	19 (51.3%)	18 (48.7%)	
>1 year	18	12 (66.6%)	6 (33.4%)	

CNY: China Yuan. Travelling time to hospital: The travel time that one patient spends on journey from home to hospital. Out-of-pocket costs: The per-hospitalization expenses that patients must bear, excluding insurance reimbursements. Social insurance: Social insurance is a fundamental insurance provided by the state, but the reimbursement amount and scope of medications are limited. Commercial insurance: Commercial insurance is a supplementary insurance operated by insurance companies on top of Social insurance. It offers a larger reimbursement amount and broader scope of medications compared to social insurance.

### Financial toxicity

**Figure 2:**
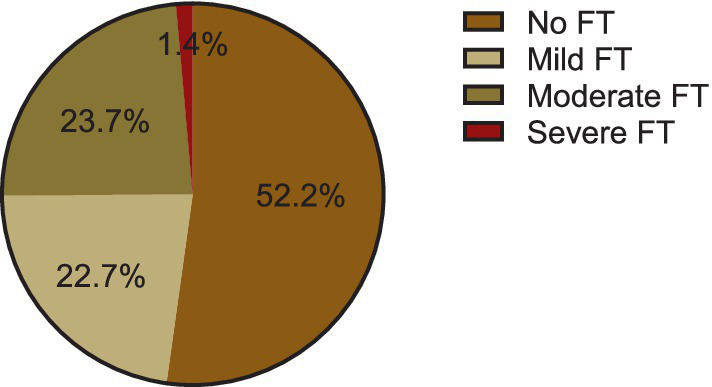
Prevalence of financial toxicity in patients with DTC.

The mean COST score was 22.4 (SD 10.6). The prevalence of financial toxicity was 47.8% (95% CI: 41% ~ 54.7%). Of these, 22.7% (47/207) were mild FT, 23.7% (49/207) were moderate FT, and 1.4% (3/207) were severe FT. The distribution of COST score and FT severity can be seen in Figure 2.

### Variables associated with financial toxicity

The univariate analysis of baseline variables associated with financial toxicity was described in Table 2. In univariate analysis, patients reporting financial toxicity tended to be younger, female, live in rural areas, have lower educational levels, be uninsured, have lower income, longer travel time, higher out-of-pocket (OOP) costs, and advanced cancer (Table 2). After adjusting for potentially confounding variables in the multivariable modeling, the following factors were associated with increased financial toxicity: younger age (odd ratio [OR], 4.66; *p* = 0.016), lower educational level (OR, 2.59; *p* = 0.011), uninsured status (OR, 3.29; *p* = 0.048), lower household income (OR, 6.34; *p* = 0.024), higher OOP costs (6,000 ~ 10,000 CNY: OR, 4.11; *p* = 0.003; >10,000 CNY: OR, 7.13; *p* = 0.001), and advanced cancer (OR, 3.99; *p* = 0.004).

**Table 2:** Univariable and multivariable logistic regression models predicting the likelihood of self-reported financial toxicity.

Characteristics	Univariate analysis		Multivariable analysis	
	OR (95% CI)	*p*-value	OR (95% CI)	*p*-value
Age				
>55	Reference		Reference	
40 ~ 55	4.28 (1.90–9.65)	**0.001**	3.89 (1.79–12.09)	**0.002**
20 ~ 40	4.68 (2.06–10.91)	**0.001**	4.66 (1.29–11.71)	**0.016**
Sex				
Male	Reference		Reference	
Female	1.48 (0.83–2.63)	**0.026**	1.13 (0.52–2.43)	0.756
Place of residence				
Urban	Reference		Reference	
Rural	1.88 (1.08–3.27)	**0.001**	1.39 (0.75–3.14)	0.336
Marital status				
Married	Reference		Reference	
Unmarried	1.08 (0.51–2.26)	0.841	1.42 (0.55–3.66)	0.458
Divorced	2.81 (0.93–8.57)	0.066	2.89 (0.62–13.51)	0.176
Widowed	5.12 (1.05–24.97)	0.042	2.20 (0.32–15.10)	0.422
Education level				
College graduated or high (>12 year)	Reference		Reference	
High school or lower (<9 year)	4.32 (1.87–9.92)	**0.001**	2.59 (1.15–4.33)	**0.011**
Some college (9 ~ 12 year)	4.03 (1.66–9.80)	**0.002**	2.02 (1.43–5.81)	**0.008**
Employment status				
Employed	Reference		Reference	
Unemployed	0.95 (0.40–2.26)	0.951	1.84 (0.83–4.08)	0.134
Retired	2.25 (0.92–5.47)	0.074	1.54 (0.44–3.20)	0.499
Health insurance				
Social and Commercial insurance	Reference		Reference	
Social insurance	3.19 (1.49–11.72)	0.002	1.67 (1.79–12.84)	**0.033**
Uninsured	5.96 (2.83–28.28)	**0.001**	3.29 (1.02–5.01)	**0.048**
Household income (CNY)				
>300,000	Reference		Reference	
200,000 ~ 300,000	5.35 (1.56–18.31)	**0.007**	5.13 (1.37–13.32)	**0.015**
120,000 ~ 200,000	6.02 (1.85–19.62)	**0.003**	4.13 (1.31–19.16)	**0.017**
60,000 ~ 120,000	7.21 (2.21–23.44)	**0.041**	5.86 (1.33–21.87)	**0.019**
<60,000	15.62 (4.10–59.46)	**0.001**	6.34 (1.12–30.84)	**0.024**
Out-of-pocket costs (CNY)				
<6,000	Reference		Reference	
6,000 ~ 10,000	5.65 (3.56–9.31)	**0.017**	4.11 (1.37–13.32)	**0.003**
>10,000	9.02 (5.15–18.62)	**0.001**	7.13 (4.32–14.93)	**0.001**
Travelling time to hospital				
<30 min	Reference		Reference	
30 min ~ 1 h	0.93 (0.30–2.82)	0.903	0.63 (0.13–3.13)	0.579
1 ~ 2 h	2.13 (0.84–5.43)	0.110	0.97 (0.16–5.74)	0.972
2 ~ 5 h	6.68 (2.45–18.20)	**< 0.001**	3.55 (0.55–22.96)	0.184
Tumor stage				
I ~ II	Reference		Reference	
III ~ IV	12.66 (5.81–27.40)	**0.003**	3.99 (1.58–10.24)	**0.004**
Number of chronic disease				
0	Reference		Reference	
1	1.79 (0.78–4.09)	0.165	1.02 (0.35–2.92)	0.975
≥2	1.17 (0.45–3.00)	0.746	1.36 (0.42–4.42)	0.603
Diagnosis time				
<1 month	2.88 (0.91–9.12)	0.070	2.81 (0.65–12.07)	0.163
1 ~ 6 month	1.66 (0.58–4.75)	0.344	1.56 (0.41–5.88)	0.042
6 ~ 12 month	1.89 (0.58–6.12)	0.286	1.15 (0.24–5.32)	0.859
>1 year	Reference		Reference	

Bold values indicate statistical significance (*p* < 0.05).

### Financial toxicity and psychological distress

**Figure 3:**
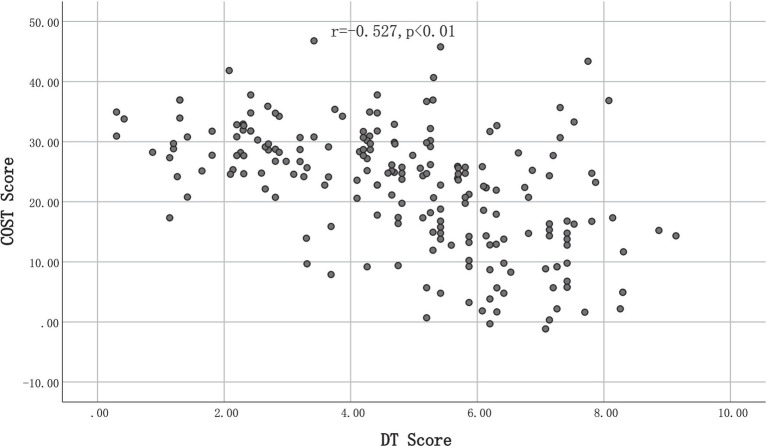
Correlation between financial toxicity and psychological distress.

The mean score for psychological distress, as measured by the DT, was 4.84 with a standard deviation of 2.04, across the entire study population. The Pearson correlation coefficient (r) between the COST score and DT score was −0.56 (*p* < 0.001), indicating a strong correlation between financial toxicity and psychological distress. However, after adjusting for potential confounding variables such as age, gender, and education, the correlation coefficient between COST and DT decreased to −0.527, with no significant change in the overall relationship. Figure 3 illustrated that as the COST score decreases, the DT score increases accordingly.

## Discussion

To the best of our knowledge, this is the first Financial toxicity (FT) survey in the context of thyroid cancer conducted in China. Our study revealed that FT was observed in 47.8% of patients with DTC, with 22.7, 23.7, and 1.4% in grade 1, grade 2, and grade 3, respectively. The findings suggest that patients with DTC experienced less FT than previously reported in FT surveys conducted on Chinese populations (17–22). For instance, Jiang et al. (17) reported an FT prevalence of 66.2% among nasopharyngeal carcinoma patients, with 37.1% experiencing mild FT, 50.5% moderate FT, and 2.4% severe FT. Liu et al. (20) found that 77% of lung cancer patients had FT, including 54.5% with mild FT and 22.5% with moderate or severe FT. Similarly, Mo et al. Observed (21) an FT prevalence of 52.8% among colorectal cancer patients, while Jing et al. reported that approximately 50% of breast cancer patients in China experienced FT. (22)This difference is partly attributed to cancer type, as DTC is generally less costly to treat in China compared to other cancer (23). Additionally, patients with DTC often have better prognoses that enable them to keep working after surgery (24). Evidence indicates that patients’ work status is strongly linked to their FT, and those who continue working are less likely to report FT than those who do not due to their illness (25).

The findings of this study demonstrate the potential and applicability of utilizing a survey based on the COST scale for assessing the FT linked with the management of thyroid cancer. The outcomes of the survey indicate that FT is a prevalent issue among individuals with DTC. By conducting a multifactorial regression analysis and controlling for potential confounders, our results reveal a significant association between FT and several factors, including younger age, lower educational attainment, lack of insurance, lower household income, and advanced cancer stage. Compared to other forms of cancer, DTC tends to manifest at a younger age (26). Our study corroborates previous research, demonstrating that younger age is associated with a higher likelihood of FT. (27, 28) The majority of patients included in our investigation were from the western region of China, which may be attributed to the relatively lower economic development in this area, resulting in a lower average income compared to the eastern region. A study conducted by Han et al. (29) involving 963 cancer survivors who completed the 2016 MESP experiences with Cancer self-administered questionnaire (SAQ) discovered that younger survivors between the ages of 18 to 64 years were more likely to experience financial sacrifices as a result of cancer, as compared to survivors over 65 years of age. Hazell et al. (30) also found that younger individuals have lower levels of financial savings, which is associated with higher FT. This may be due to the fact that younger patients have less time to accumulate financial assets and carry greater financial obligations, such as housing loans, which may exacerbate their financial toxicity (31). In addition, Chinese culture places great emphasis on supporting the older adult (32), with a previous study indicating that (33) over 50% of the medical expenses incurred by older adult Chinese individuals are borne by their children. Thus, a diagnosis of thyroid cancer may further exacerbate the already significant financial burden experienced by many families. Interestingly, although youth tends to be associated with higher FT, 44% of the patients in this study were between the ages of 20–40 years, which may be explained by the fact that the patients included in this study were patients with DTC.

Additionally,out-of-pocket (OOP) costs also played a significant role in FT. Our study found that higher OOP expenses were significantly associated with increased FT. Specifically, compared to patients with OOP costs below 6,000 CNY, those with OOP costs between 6,000 and 10,000 CNY had a significantly higher likelihood of experiencing FT (*p* = 0.003), while those with OOP costs exceeding 10,000 CNY exhibited an even greater probability (*p* = 0.001). These findings suggest that OOP medical expenditures remain a crucial component of the overall financial burden for cancer patients, despite the extensive coverage of China’s healthcare system. It is noteworthy that China’s social medical care system, including the Urban Employees’ Basic Medical Insurance (URBMI) and the Urban and Rural Residents’ Basic Medical Insurance (URRBMI), covers over 1.3 billion individuals, accounting for more than 95% of the population (33). Despite this extensive coverage, our study revealed that patients with DTC were least likely to experience FT when covered by both social and commercial insurance, rather than either type of insurance alone. This result suggests that cost-sharing plays a crucial role in mitigating FT. However, even with commercial health insurance, China’s healthcare system only covers hospital-related treatment costs, such as hospitalization and medication costs, but not transportation, accommodation, and specialist consultation expenses. Michael et al. (9) reported that lacking insurance is not only a significant risk factor for FT but also associated with lower quality of life and mortality in patients with atopic disease. Mejrl et al. (34) highlighted the importance of conducting FT surveys in low- and middle-income areas and for high-burden cancers because these patients are the most vulnerable to FT. Financial constraints may lead to a reduction in necessary tests and medications or even treatment abandonment. FT is expected to be more severe in low- and middle-income areas due to the direct correlation between economic toxicity and the inability to afford medical care (35). Beeler et al. (36) further demonstrated that lower-income households are associated with a more severe financial burden. Despite the rising costs associated with innovative therapies, such as immune and targeted therapies (37), the primary treatment measures for DTC in China are surgery and radiation therapy (38). Most of our patients underwent only surgical treatment, with an FT score of 22.4, higher than those who mainly received radiation therapy with an FT score of 21.9 (39), suggesting lower economic toxicity. Fabian et al. (40) reported that receiving radiation therapy was associated with a higher out-of-pocket financial burden of costs. The existing economic development level and per capita income in western China, in comparison to other regions, primarily manifests in the following three dimensions. Firstly, in contrast to other regions, the rural economy in western China exhibits a lower degree of advancement. Secondly, western China accommodates a significant populace of ethnic minorities. As per the seventh national population census carried out in 2020, 70.2% of the ethnic minority population concentrates in western China (39). Concurrently, these ethnic minorities predominantly reside in remote mountainous areas, characterized by austere natural surroundings, unfavorable health conditions, and elevated disease prevalence (19). Thirdly, denizens of rural areas in the western region experience restricted access to health resources and medical services in comparison to their counterparts in the eastern and central regions (19).

This study reveals that increased financial toxicity in patients with DTC is associated with greater psychological distress, consistent with existing literature on financial toxicity in cancer care. Margaret (46)reported a correlation between psychological distress and financial toxicity in women with breast cancer, regardless of whether they experienced financial toxicity within 5–25 months of diagnosis. The impact of financial toxicity on patient and family decision-making related to spending on leisure activities, basic items, savings, work hours, and ability to return to work may account for this association (40). The ability of healthcare professionals to identify and intervene regarding FT may have both short- and long-term effects on patients’ psychological well-being. In this study, patients with higher FT had higher psychological distress, as indicated by the negative correlation between COST scores and DT scores (41). Patients with low financial reserves also reported increased pain, which was similarly noted by Christopher (42), who observed that patients with FT had more severe anxiety and depression at baseline and showed less improvement over time compared to those without FT. Thus, FT may serve as a significant source of distress for patients. Healthcare professionals should measure patients’ levels of FT during the admission-to-discharge follow-up process, with a focus on analyzing the prevalence and specificity of the problem among patients from different cultural backgrounds. For instance, Blayney et al. (43) conducted research with the aim of identifying high-quality and cost-effective care options for cancer patients. By assessing total expenditures to the quality of care provided, the study deduced four essential themes for enhancing care quality. These include optimizing treatment planning and goal-setting, providing comprehensive navigator and palliative care services, fostering a highly skilled care team, and establishing a robust infrastructure. These measures are envisioned to improve the quality of patients’ survival while concurrently alleviating their financial burdens. Simultaneously, healthcare professionals ought to address the concern of FT by implementing technological solutions, such as automated software systems, to gauge patients’ financial situations accurately. Whenever healthcare professionals encounter unresolved FT during clinical interactions, they should promptly refer patients to social work and financial navigation services to facilitate tailored FT management (44). Nevertheless, research has shown that cancer patients often perceive healthcare professionals as insufficiently attuned to their financial needs. This suggests the necessity for further refinement of financial hardship screening processes, with a view to effectively integrating financial toxicity assessment with essential counseling, navigation, and referral services (45). Thus, future endeavors should concentrate on optimizing and mitigating FT in patients by actively engaging healthcare professionals and fostering strong support and collaboration among multidisciplinary team members. This will facilitate the exploration of solutions to FT.

## Limitations

Our study has several limitations. First, the data on out-of-pocket (OOP) costs were self-reported by patients, which may introduce recall bias. Patients may have had difficulty accurately recalling the full extent of their OOP expenses, particularly those incurred over a prolonged treatment period, potentially affecting the accuracy of the data. Second, in our multivariable analysis, some odds ratios exhibited wide confidence intervals, likely due to limited sample sizes in certain subgroups or variability in patient characteristics. Third, as our study utilized a cross-sectional design, it cannot establish causality between financial toxicity and psychological distress. Future longitudinal studies are needed to explore the temporal relationship and causality between financial burden and patient outcomes. Lastly, since the study was conducted in two tertiary hospitals in western China, the generalizability of the findings may be limited. Future multi-center studies across different regions of China are warranted to provide more comprehensive insights.

## Conclusion

In this study, the prevalence of FT was 47.8% and psychological distress was prevalent among patients with DTC. Although the prognosis for patients with this type of cancer is generally favorable, the findings of this study underscore the need to identify patients with high FT and to provide appropriate support. Future research should focus on identifying risk factors associated with FT and exploring the interaction between FT and psychological distress. These efforts will help to identify patients at risk and develop targeted interventions to mitigate the negative effects of FT on patient well-being.

## Data Availability

The raw data supporting the conclusions of this article will be made available by the authors, without undue reservation.
